# Shock index in patients with traumatic solid organ injury as a predictor of massive blood transfusion protocol activation

**DOI:** 10.1186/s40621-019-0218-7

**Published:** 2019-10-07

**Authors:** Ayman El-Menyar, Gaby Jabbour, Mohammad Asim, Husham Abdelrahman, Ismail Mahmood, Hassan Al-Thani

**Affiliations:** 1 Clinical Medicine, Weill Cornell Medical College, Doha, Qatar; 2 Department of Surgery, Clinical Research, Trauma & Vascular Surgery, Hamad General Hospital (HGH), P.O Box 3050, Doha, Qatar; 3 Department of Surgery, Trauma Surgery, HGH, Doha, Qatar; 4 Department of Surgery, Trauma & Vascular Surgery, HGH, Doha, Qatar

**Keywords:** Shock index, Solid organ injury, Blood transfusion, Laparotomy, MTP, Trauma

## Abstract

**Purpose:**

We aimed to assess the utility of shock index (SI) to predict the need for massive transfusion protocol (MTP) in patients with solid organ injury (SOI) in a Level 1 Trauma center.

**Methods:**

We conducted a retrospective analysis for patients with SOI between 2011 and 2014. Patients were categorized according to on-admission SI into low (< 0.8) and high SI (≥0.8) group.

**Results:**

A total of 4500 patients were admitted with trauma, of them 572 sustained SOIs (289 patients had SI ≥0.8). In comparison to low SI, patients with high SI were younger, had higher injury severity scores (ISS) and lower Trauma and Injury Severity Score (TRISS); (*p* < 0.001). The proportion of exploratory laparotomy (EXLap), blood transfusion (BT), MTP activation, sepsis and hospital mortality were significantly higher in patients with high SI. Serum lactate (*r* = 0.34), hematocrit (*r* = − 0.34), ABC score (*r* = 0.62), ISS (*r* = 0.35), and amount of transfused blood (*r* = 0.22) were significantly correlated with SI. On multivariable regression analysis using 9 relevant variables (age, sex, ISS, ED GCS, serum lactate, hematocrit, Abdomen AIS and Focused assessment with sonography in trauma (FAST) and SI), SI ≥ 0.8 was an independent predictor of BT (OR 2.80; 95%CI 1.56–4.95) and MTP (OR 2.81;95% CI 1.09–7.21) .

**Conclusions:**

In patients with SOI, SI is a simple bedside predictor for BT and MTP activation. Further prospective studies are needed to support our findings.

## Introduction

In an attempt to identify hypovolemic shock in trauma, shock index (SI) has been used as a quick bedside clinical indicator of hypovolemic shock (McNab et al. 2013). It can reliably identify hemodynamic instability (Cannon et al. 2009; Vandromme et al. 2011), and could be used for risk stratification for transfusion requirements and outcomes (Zarzaur et al. 2008). Prior studies found that prehospital and admission SI correlated with on-going bleeding and need for massive transfusion (MT) in trauma patients (Vandromme et al. 2011; Zarzaur et al. 2008; Birkhahn et al. 2005; El-Menyar et al. 2018). Polytrauma patients are frequently diagnosed to have abdominal injuries with an estimated frequency of 15–17/100,000 in Qatar (Arumugam et al. 2015). In particular, abdominal solid organ injury (SOI) secondary to high-impact trauma results in considerable bleeding, morbidity and mortality (El-Menyar et al. 2017). SOI includes any grade of injury to the liver, spleen, kidneys or pancreas in isolation or combination. SOI is a leading cause of mortality in trauma and its management and outcomes are mainly dependent on the patient hemodynamic stability and the early efficient control of bleeding. Timely determination of the need for MT and intervention remains challenging in patients with SOI. Rapid diagnosis of SOIs, mainly liver and spleen, is important to minimize the risk of hemorrhagic shock, the need for surgery and post-operative complications (Sawhney et al. 2014). Abdominal trauma patients who are found to have lower grade SOI with normal physiological status are usually treated non-operatively (El-Menyar et al. 2017). On the other hand, hemodynamic instability despite resuscitation is an indication to consider immediate exploratory laparotomy and definitive surgery (Stawicki 2017). Also, it has been suggested that hemodynamically unstable patients or those who need > 2 units of packed RBC transfusion following SOI require immediate laparotomy (Malhotra et al. 2002).

It has been noted that trauma patients who require MT often die within 6 h of the resuscitation efforts, therefore, a reliable tool to predict MT usage would be vital. There are almost 24 scoring tools and predictive models available to predict the need for MT in trauma cases. Although MT is a life-saving treatment, it can be a source of harm when it is utilized inappropriately. An optimized transfusion strategy with appropriate blood component selection is critical in the absence of the point of care testing. The resuscitative effort should start within minutes (Hsu et al. 2016), however, the compliance to MT protocols (MTP) is not optimum in reality. Bawazeer et al. found delays in 50% of MTP activation and a 47% incidence of non-compliance with the type of blood product given (Fredericks et al. 2017). Notably, clinical gestalt is an unreliable predictor of MT with a sensitivity of only 66%; it worked poorly as a screening test for MT and missed over one third of patients who ultimately required MT (El-Menyar et al. 2019). This means that trauma surgeons’ threshold for MTP activation is still questionable as they missed a substantial number of cases that were potentially under resuscitated (El-Menyar et al. 2019).

Despite advancement in resuscitation, hemorrhagic shock still accounts for almost one third of all trauma-related preventable deaths (Rossaint et al. 2016). Improved prediction of significant traumatic hemorrhage may be useful for better management of blood products, and proper activation of MTP. This would reduce blood product use, and allows components of a MTP to be delivered in a timely fashion and in a high ratio [a higher fresh frozen plasma (FFP)/packed red blood cell (RBC) ratio] to treat acute traumatic coagulopathy (Khan et al. 2013).

Although SOI is one of the main sources of massive bleeding in abdominal injuries in both blunt and penetrating trauma, less is known about the prognostic implications of SI in traumatic SOI. Therefore, the current study aims to assess the utility of SI in patients with SOI, to predict the need for MTP activation, blood transfusion and exploratory laparotomy in a level I trauma center.

## Methods

This was a retrospective chart review study to include all abdominal trauma patients with SOI (splenic, hepatic, renal or pancreatic injury of any grade) admitted at the level I trauma center, between June 2011 and June 2014. Relevant information was abstracted from The Qatar National Trauma Registry [QNTR] at Hamad General Hospital (HGH) after obtaining the ethical approval from the Medical Research Center (IRB# 14409/14) at Hamad Medical Corporation. The QNTR is a database that participates in both the National Trauma Data Bank (NTDB) and the Trauma Quality Improvement Program (TQIP) of the American College of Surgeons-Committee on Trauma (ACS-COT).

**Shock Index (SI)** is defined as the ratio of HR to SBP on-admission at the emergency room, and we used the cutoff of 0.8 indicated for the need of MT in trauma patients as mentioned in an earlier study (El-Menyar et al. 2018). Prior study used this cutoff based on the optimum reading on the receiver operating characteristic curve (ROC). The pulse pressure (PP) was calculated as the difference between SBP and diastolic blood pressure (DBP).

We included all adult patients who were transported directly to the Emergency Department (ED) from the scene.

We excluded patients who were transferred from other hospitals, patients with prehospital cardiac arrest, and those who had incomplete data. The variables of interest were patient demographics (age & gender), type and mechanism of injury (MOI), vital signs on admission (i.e., HR, SBP, DBP, PP), laboratory findings (lactate, hematocrit), admission Glasgow Coma scale (GCS), Injury Severity Score (ISS), Abdomen Abbreviated Injury Scale (AIS), Trauma and Injury Severity Score (TRISS), Assessment of Blood Consumption (ABC) score, site of injury (liver, spleen, kidney & pancreas), associated injuries (head injury, hemothorax); Focused Assessment with Sonography in Trauma (FAST), intubation, exploratory laparotomy, need for blood transfusion, number of transfused packed red blood cells (PRBC) units, massive transfusion, hospital and ICU lengths of stay, ventilatory days, sepsis and mortality. SOI grading was according to the Organ Injury Scaling Committee of the American Association for the Surgery of Trauma. The American College of Surgeons has defined four classes of hypovolemic shock in the Advanced Trauma Life Support (ATLS) training program and manual; based on estimated blood loss, vital signs (blood pressure, pulse rate), and mental status. ABC score was calculated using 4 variables i.e. MOI (penetration = 1, blunt = 0), FAST (positive = 1, negative = 0), ED SBP (< 90 mmHg = 1,> 90 mmHg = 0), and ED HR (> 120 beats/min = 1, < 120 beats/min = 0) (Schroll et al. 2018). As per the institutional protocols, Massive transfusion (MT) is defined as the replacement of the patient’s total blood volume (approximately 5 l) over a 24 h period or actual/anticipated administration of > 40 mL/kg PRBC in 2 h or less. The attending physician, trauma team leader, consultant or anesthetist is responsible for activation of the MTP. Blood bank staff will immediately prepare the first pack of blood products as 6 units of uncross-matched type O positive PRBC; 6 units equivalent of platelets; and 6 units AB plasma.

### Statistical analysis

Data were presented as proportions, medians, or mean ± standard deviation, as appropriate. The variables of interest were compared and analyzed according to SI in ED (SI < 0.8 versus SI ≥ 0.8). The SI cutoff of 0.8 was used based on previous works (El-Menyar et al. 2018). Prior study used this cutoff based on the optimum reading on the receiver operating characteristic curve (ROC). Differences in categorical and continuous variables were analyzed using χ^2^ test and students t-test, as appropriate. Yates’ corrected chi-square was used for categorical variables, if the expected cell frequencies were below 5. The Pearson correlation coefficient (r) was calculated to identify the linear relationship between the SI and other relevant covariates. Predictive value of SI for MTP, blood transfusion, exploratory laparotomy and mortality was performed in terms of sensitivity, specificity, positive and negative predictive value (PPV&NPV), positive and negative likelihood ratio (LR). Multivariable regression analyses were performed to determine the predictors of blood transfusion, MTP and exploratory laparotomy using the most relevant covariates: sex, age, ISS, abdomen AIS, ED GCS, serum lactate, hematocrit, FAST and SI (SI was used as categorical in one analysis and as a continuous variable in another analysis). For prediction of early laparotomy, MTP activation as an independent variable was added into the model in addition to the above-mentioned 8 variables. Data were expressed by the odds ratio (OR) and 95% confidence intervals (CIs). SI was used as categorical and also as a continuous variable in the multivariable analysis. A two-tailed *P* value of < 0.05 was considered to be statistically significant. The Receiver Operating Characteristic (ROC) curves and area under the curves (AUC) were performed using different SI cut-offs to show the prediction power of SI for blood transfusion. All data analyses were carried out using the Statistical Package for the Social Sciences, version 18 (SPSS, Inc., Chicago, IL).

## Results

During the 3-years study duration, a total of 4500 trauma patients required hospital admission, of which 572 (12.7%) sustained SOIs. The mean age of patients was 29 ± 13.0 years and males (89%) predominated with a male to female ratio of 8 to 1. Blunt trauma (95%) was most frequent injury type which constituted mainly motor vehicle crashes (78%) and fall from height (22%). On arrival to the trauma room, 289 (50.5%) SOI patients had an elevated SI (≥0.8) (Table 1).

**Table 1:** Clinical characteristics, presentation and outcome by shock index in abdominal trauma patients sustained solid organ injuries

	Overall (*n* = 572)	SI < 0.8 (*n* = 283)	SI ≥0.8 (*n* = 289)	*P* value
Age (mean ± SD)	29.2 ± 13.0	32.4 ± 12.5	26.1 ± 12.9	0.001
Males	509 (89.0%)	263 (92.9%)	246 (85.1%)	0.003
Blunt trauma	543 (94.9%)	264 (93.3%)	279 (96.5%)	0.07 for all
Penetrating injuries	29 (5.1%)	19 (6.7%)	10 (3.5%)	
Mechanism of injury				
Motor vehicle crash	365 (77.7%)	170 (73.6%)	195 (81.6%)	0.03 for all
Fall from height	105 (22.3%)	61 (26.4%)	44 (18.4%)	
Pulse pressure ED	46.6 ± 16.6	51.9 ± 15.8	41.3 ± 15.6	0.001
Initial lactate	2.9 (0.6–23.4)	2.48 (0.8–23.4)	3.4 (0.6–22.5)	0.001
Second lactate	3.0 (0.7–80.0)	2.7 (0.7–39.0)	3.2 (0.8–80.0)	0.03
Initial hematocrit	39.6 ± 7.0	41.2 ± 6.0	38.0 ± 7.6	0.001
Second hematocrit	36.6 ± 6. 45	37.9 ± 5.7	35.3 ± 7.0	0.001
GCS ED	12.4 ± 2.6	13.9 ± 1.1	10.9 ± 4.1	0.001
Injury Severity Score	18.7 ± 10.7	15.0 ± 8.1	22.2 ± 11.7	0.001
Abdomen AIS	2.6 ± 0.9	2.4 ± 0.7	2.7 ± 0.9	0.001
TRISS	0.9618 ± 0.0873	0.9822 ± 0.0457	0.9381 ± 0.1143	0.001

Shock Index (SI) = pulse ED / SBP ED; Pulse pressure = SBP ED - DBP ED; GCS: Glasgow Coma score

In comparison to lower SI, patients with elevated SI were 6 years younger (26 ± 13 vs 32 ± 12.5, *p* = 0.001), had lower PP (41.3 ± 16 vs 52 ± 16, *p* = 0.001), lower GCS (11 ± 4 vs 14 ± 1, *p* = 0.001), lower TRISS (0.9381 ± 0.114 vs 0.9822 ± 0.045, *p* = 0.001), and lower hematocrit values. Patients with elevated SI also had elevated initial serum lactate levels (median; 3.4 vs. 2.5, *p* = 0.001), greater ISS (22 ± 12 vs 15 ± 8.0, *p* = 0.001) and higher abdominal AIS (2.7 ± 0.9 vs 2.4 ± 0.7, *p* = 0.001). The mean SI was relatively higher in those who had blunt in comparison to penetrating trauma (0.89 ± 0.36 vs 0.79 ± 0.30).

**Fig. 1:**
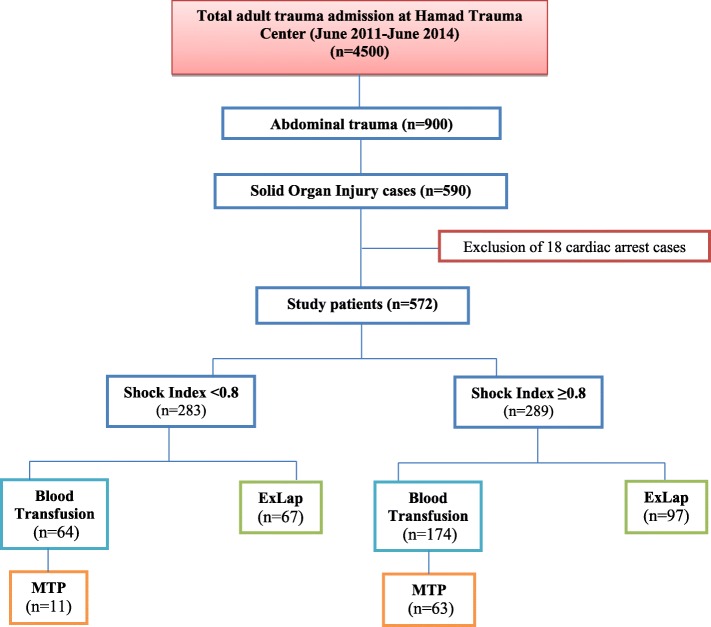
Flow chart of the study design (MTP: Massive Transfusion Protocol; ExLap: Exploratory laparotomy)

Table 2 compares the associated injuries, hospital course and outcome by SI. The two groups were comparable for the reported SOI and hemothorax (*p* > 0.05 for all). The elevated SI group showed higher association with head injury (35.6% vs. 16.6%, *p* = 0.001) and retroperitoneal hematoma (10.4% vs. 4.2%, *p* = 0.005). The rate of exploratory laparotomy (33.6% vs. 23.7%, *p* = 0.009), blood transfusion (60.2% vs. 22.6%, *p* = 0.001) and MTP activation (21.8% vs. 3.9%, *p* = 0.001) were significantly higher in patients with elevated SI. Figure 1 shows the study design and outcome.

**Table 2:** Associated injuries and outcome by shock index

	Overall (*n* = 572)	SI < 0.8 (*n* = 283)	SI ≥0.8 (*n* = 289)	*P* value
Solid Organ injury^a^				
Liver	289 (50.5%)	143 (50.5%)	146 (50.5%)	0.99
Splenic	211 (36.9%)	97 (34.3%)	114 (39.4%)	0.20
Kidney	122 (21.3%)	59 (20.8%)	63 (21.8%)	0.78
Pancreas	32 (5.6%)	12 (4.2%)	20 (6.9%)	0.16
Associated injuries				
Head injury	150 (26.2%)	47 (16.6%)	103 (35.6%)	0.001
Hemothorax	53 (9.3%)	24 (8.5%)	29 (10.0%)	0.52
Retroperitoneal hematoma	42 (7.3%)	12 (4.2%)	30 (10.4%)	0.005
Pelvic hematoma	16 (4.2%)	5 (2.7%)	11 (5.7%)	0.14
Mesenteric injury	53 (9.7%)	27 (9.9%)	26 (9.4%)	0.83
FAST Positive	147 (27.8%)	61 (23.6%)	86 (31.7%)	0.03
Intubation	209 (36.5%)	48 (17.0%)	161 (55.7%)	0.001
Exploratory laparotomy	164 (28.7%)	67 (23.7%)	97 (33.6%)	0.009
Blood transfusion	238 (41.6%)	64 (22.6%)	174 (60.2%)	0.001
Blood unit transfused	6 (1–62)	4 (1–62)	6.5 (1–51)	0.001
MTP activation	74 (12.9%)	11 (3.9%)	63 (21.8%)	0.001
ICU LOS	4 (1–76)	3 (1–76)	5 (1–69)	0.003
Ventilatory days	3 (1–32)	2 (1–32)	3 (1–31)	0.48
Hospital LOS	8 (1–304)	6 (1–122)	10 (1–304)	0.001
Sepsis	35 (6.1%)	7 (2.5%)	28 (9.7%)	0.001
In-hospital mortality	50 (8.7%)	9 (3.2%)	41 (14.2%)	0.001

^a^The frequency of each solid organ is overlapping between the four organs; *SI* Shock Index, *MTP* Massive transfusion protocol, *LOS* Length of stay

**Fig. 2:**
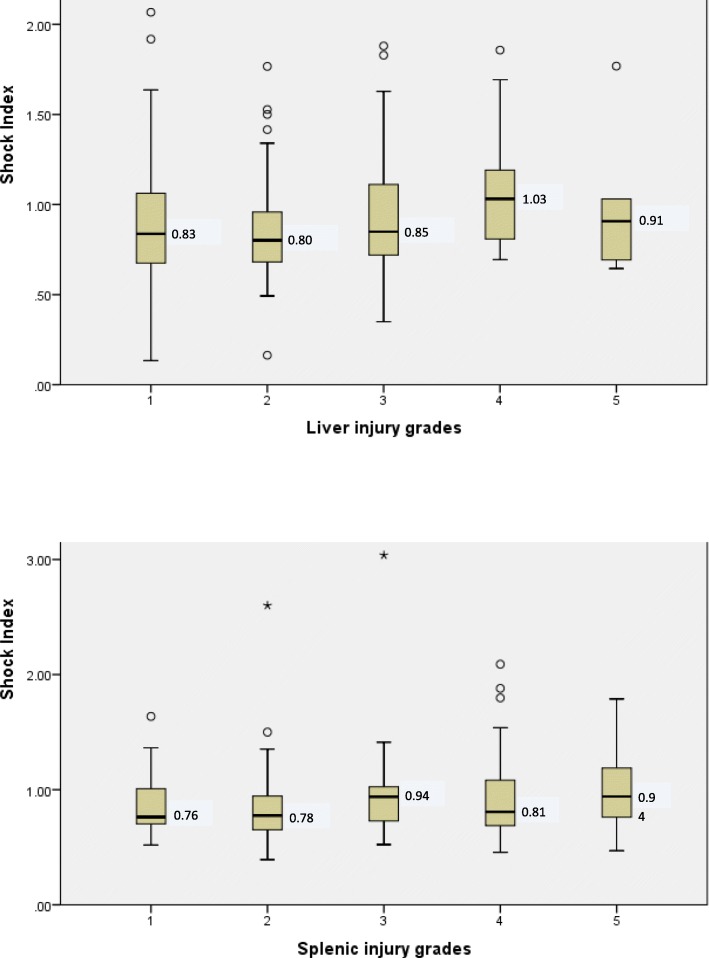
Shock Index by (**a**) liver and (**b**) splenic injury grades

Amongst the different injured organ, SI values varied and was higher in the injured organ in comparison to the non-injured organ; for example: it was 0.94 ± 0.35 in pancreatic injury vs 0.88 ± 0.36 in non-injured pancreas), 0.92 ± 0.41 in renal injury vs 0.87 ± 0.34 in non-injured kidney, 0.91 ± 0.37 in splenic injury vs 0.86 ± 0.35in non-splenic injury and 0.89 ± 0.36 in hepatic injury vs 0.89 ± 0.35 in non-hepatic injury). Figure 2 shows that the median SI increased with the grade of liver and spleen injuries.

A significantly longer median ICU and hospital length of stay were found among patients with SI ≥ 0.8 than those with SI < 0.8. Likewise, the proportions of sepsis (9.7% vs 2.5%) and hospital death (14.2% vs 3.2%) were significantly higher in patients with SI ≥ 0.8 (almost 4 times) when compared to those with SI < 0.8.

Table 3 demonstrates a significant positive and negative correlation between SI and other predictors.

**Table 3:** Correlations between SI, clinical parameters and injury severity

	Pearson correlation (r)	*p* value
Age	−0.25	0.001
Pulse pressure	−0.45	0.001
Hematocrit	−0.34	0.001
Serum lactate	0.34	0.001
GCS ED	−0.40	0.001
Injury Severity score	0.35	0.001
Abdominal AIS	0.15	0.001
ABC score	0.62	0.001
TRISS	−0.24	0.001
Blood units transfused	0.22	0.001
Hospital LOS	0.23	0.001

Table 4 shows the performance and accuracy of different SI values (≥ 0.8, ≥ 0.9, and ≥ 1.0) as well as the ABC score with respect to mortality, blood transfusion, MTP and exploratory laparotomy. SI ≥ 0.8 had a higher sensitivity (85 and 82%) and negative predictive value (96 and 97%) with a negative LR of 0.27 and 0.34 to identify the need for MTP and risk of mortality, respectively.

**Table 4:** Predictive value of scoring systems

	MTP	Blood transfusion	Exploratory Laparotomy	Mortality
Shock Index (≥0.8)				
Sensitivity	85.1%	73.1%	59.1%	82%
Specificity	54.6%	65.6%	52.9%	52.5%
Positive Predictive value	21.8%	60.2%	33.6%	14.2%
Negative Predictive value	96.1%	77.4%	76.3%	96.8%
Positive likelihood ratio	1.87	2.12	1.25	1.72
Negative likelihood ratio	0.27	0.41	0.77	0.34
Shock Index (≥0.9)				
Sensitivity	75.7%	59.7%	46.3%	70%
Specificity	70.5%	81.7%	68.9%	67.8%
Positive Predictive value	27.6%	70%	37.4%	17.2%
Negative Predictive value	95.1%	74%	76.2%	95.9%
Positive likelihood ratio	2.56	3.26	1.48	2.17
Negative likelihood ratio	0.34	0.49	0.77	0.44
Shock Index (≥1.0)				
Sensitivity	63.5%	47.9%	36%	62%
Specificity	79.1%	88.9%	77.5%	77%
Positive Predictive value	31.1%	75.5%	39.1%	20.5%
Negative Predictive value	93.6%	70.5%	75.1%	95.5%
Positive likelihood ratio	3.03	4.31	1.6	2.69
Negative likelihood ratio	0.46	0.58	0.82	0.49
ABC Score				
Sensitivity	44.9%	28.1%	32%	45.5%
Specificity	90.2%	95.7%	92.6%	88.5%
Positive Predictive value	40.8%	82.9%	63.2%	26.3%
Negative Predictive value	91.6%	64.5%	77.5%	94.7%
Positive likelihood ratio	4.58	6.53	4.32	3.95
Negative likelihood ratio	0.61	0.75	0.73	0.61

For prediction of the need for exploratory laparotomy, the 3 SI values (≥ 0.8, ≥ 0.9, and ≥ 1.0) and ABC score showed a similar NPV (77%) with poor sensitivity (32–59%), however, ABC score showed a higher specificity (93%) and PPV of 63%.

**Fig. 3:**
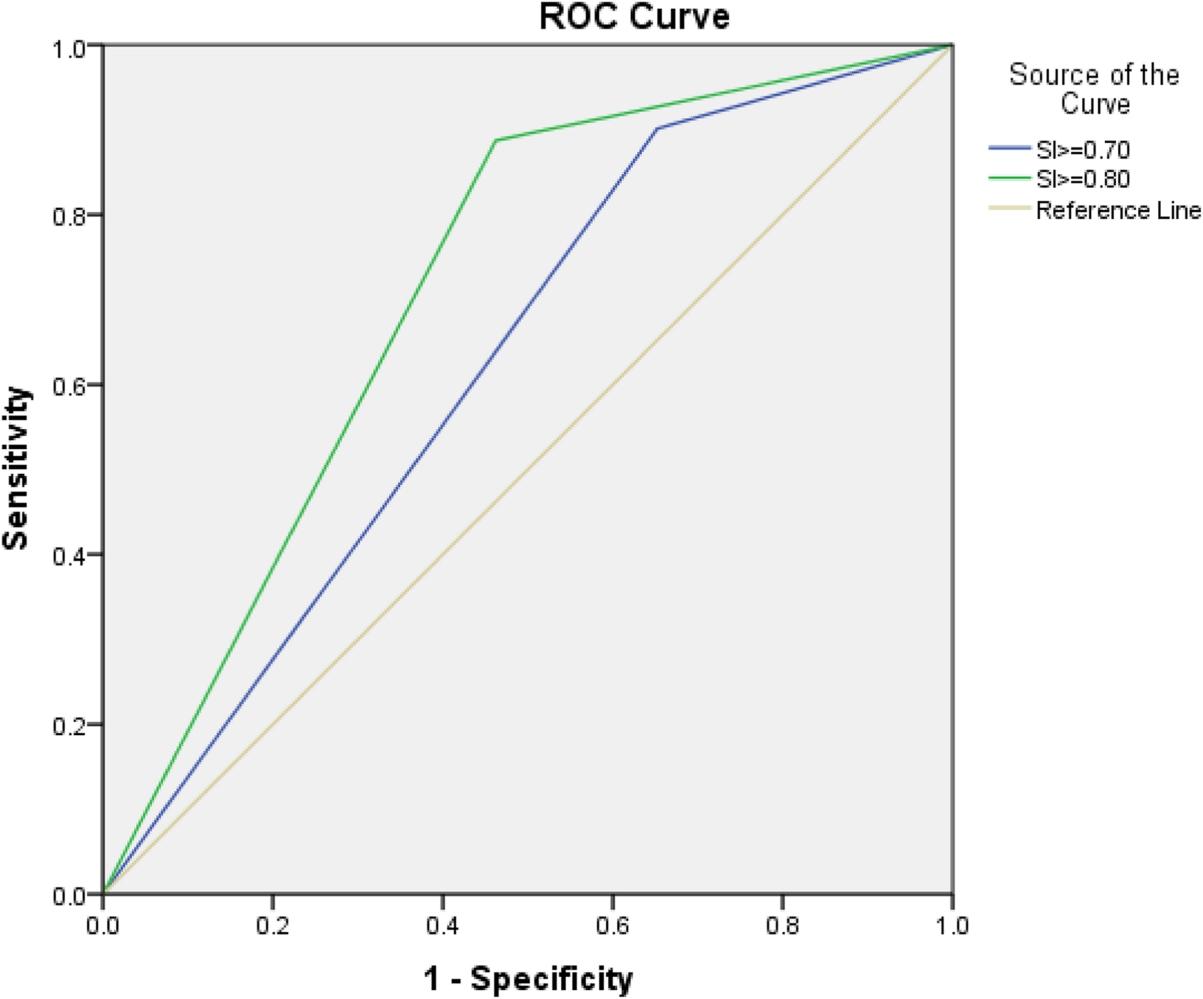
ROC curves for different shock index (SI) cut-offs for prediction of blood transfusion in solid organ injury. Area under the Curve (AUC) for SI ≥0.70 is 0.62(0.56–0.69) and 0.71(0.66–0.77) for SI ≥ 0.80

**Fig. 4:**
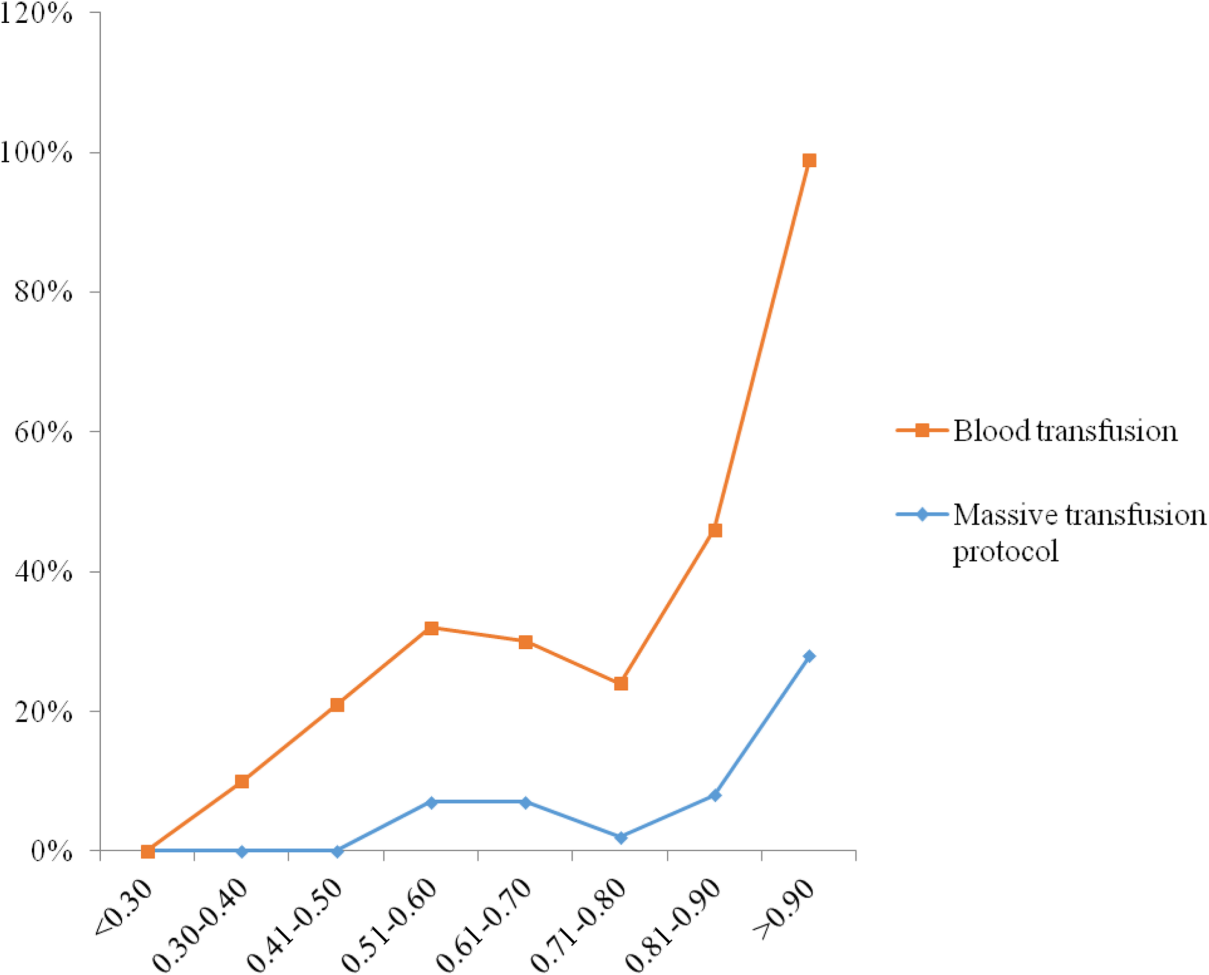
Relationship between blood transfusion, massive transfusion protocol and different shock indexcut-offs

Figure 3 shows the ROC curves for different shock index (SI) cut-offs for prediction of blood transfusion in SOI. The AUC for SI ≥0.70 is 0.62(0.56–0.69) and 0.71(0.66–0.77) for SI ≥ 0.80 (*p* = 0.001 for each). Different SI cut-offs (< 0.30, 0.30–0.40, 0.41–0.50, 0.51–0.60, 0.61–0.70, 0.71–0.80, 0.81–0.90, and > 0.90) were plotted against blood transfusion and MTP (Fig. 4). The need for blood transfusion and protocol increased significantly with SI 0.8 and above.

### Multivariable logistic regression analysis

Multivariable analysis using 8 relevant variables such as age, sex, initial serum lactate, initial hematocrit value, FAST positivity, abdominal AIS, ISS, ED GCS and SI, it showed that showed that SI ≥0.8 was an independent predictor of blood transfusion with OR 2.80; 95% CI 1.560–4.950). When SI was introduced as a continuous variable, the OR for blood transfusion was 15.00 (95% CI 4.180–53.16).

For the prediction of MTP activation, multivariable regression analysis using 8 covariates showed that SI ≥0.8 was independent predictor of MTP with OR 2.81 (95% CI 1.098–7.206). When SI was introduced as a continuous variable, the OR for blood transfusion was 1.41 (95% CI 0.540–3.694).

For early exploratory laparotomy, SI was not predictor of laparotomy (OR 1.23; 95% CI 0.689–2.212) (Table 5).

**Table 5:** Multivariable analysis for predictor of blood transfusion, massive transfusion protocol and laparotomy

Variable	Blood transfusion			Massive transfusion protocol			Exploratory laparotomy		
	P	OR	95% CI	P	OR	95% CI	P	OR	95% CI
Age; years^⁎^	0.187	1.016	0.992–1.040	0.137	1.025	0.992–1.059	0.151	1.02	0.994–1.040
Sex (male)	0.318	1.649	0.618–4.398	0.066	4.268	0.908–20.05	0.136	2.18	0.783–6.047
Initial Lactate^⁎^	0.001	1.418	1.179–1.704	0.166	1.09	0.966–1.226	0.116	1.09	0.977–1.233
Initial hematocrit^⁎^	0.001	0.862	0.816–0.911	0.001	0.884	0.833–0.938	0.009	0.94	0.900–0.985
Admission GCS^⁎^	0.015	0.913	0.849–0.983	0.010	0.897	0.826–0.975	0.015	0.92	0.854–0.983
ISS^⁎^	0.001	1.072	1.032–1.114	0.183	1.032	0.985–1.080	0.003	0.94	0.911–0.981
FAST result	0.016	2.266	1.168–4.397	0.079	2.24	0.910–5.532	0.001	4.14	2.347–7.452
Abdomen AIS^⁎^	0.020	1.659	1.082–2.544	0.172	1.40	0.862–2.295	0.001	2.53	1.719–3.732
SI^⁎^	0.001	15.00	4.180–53.16	0.482	1.41	0.540–3.694	0.758	0.87	0.372–2.057
SI ^⁎⁎^	0.001	2.80	1.560–4.950	0.031	2.81	1.098–7.206	0.479	1.23	0.689–2.212

⁎ = continuous variable, ^⁎⁎^Shock index (SI) as categorical variable ≥0.80 vs < 0.80 in a second multivariable model, *OR* Odd ratio, *CI* Confidence interval

## Discussion

The present study has several key findings. SI ≥ 0.8 is a useful bedside simple predictor for early management of massive bleeding including the MTP activation in patients with SOI. Multivariate analysis model failed to address the role of SI in predicting the need of exploratory laparotomy; however, it showed that SI was independent predictor of blood transfusion and MTP. Moreover, the study showed significant correlations between SI and ABC scoring, serum lactate, the amount of transfused blood, and ICU/ hospital length of stay. Furthermore, patients with SI ≥0.8 were found to have a higher rate of sepsis and in-hospital mortality in our cohort. Moreover, the higher the grade of organ injury, the higher the SI is in patients with liver and splenic injury. Therefore, SI could be used for early identification of SOI patients who are at risk of significant bleeding that requires massive transfusion. The AUC showed the superiority of SI 0.80 over the 0.70 cutoff for the prediction of blood transfusion in the present study.

The present study explores the prognostic implications of SI in terms of blood transfusion, MTP activation and exploratory laparotomy in patients with traumatic SOI. Several investigators have proposed different cut-off values for SI, of which SI ≥ 0.8, SI ≥ 0.9 or ≥ 1.0 has been used to predict critical bleeding in trauma patients (Cannon et al. 2009; El-Menyar et al. 2018; Schroll et al. 2018; Olaussen et al. 2014; Odom et al. 2016). The present study utilized SI ≥ 0.8 as this cut-off has higher sensitivity and NPV for prediction of blood transfusion. An earlier study suggested that the frequently used cutoff value of 0.9 has greater possibility of under-triage in patients necessitating urgent intervention (McNab et al. 2012).

Our institution is following the standard international management guidelines to treat patients with multiple trauma; many of these patients require blood transfusion (≈42%) and one-third of them necessitate MTP activation. Caring for such patients is resource-intensive task and requires specialized coordinated services in a critical and timely manner (Peralta et al. 2015).

Exploratory laparotomy was required in one-quarter of cases that sustained blunt trauma. The initial FAST examination in our series yielded negative results in 72% of the blunt and 75% of the penetrating trauma. In those who had negative FAST results, blood transfusion, MTP activation and early laparotomy were required in 35, 16 and 9% of cases, respectively.

On multivariate analysis, both high SI and positive FAST was almost having equal predictive value for transfusion and MTP. Although FAST has important diagnostic in the initial assessment of trauma patients, SI is an easy and early predictor that doesn’t require an operator skill.

A systematic review (Bruijns et al. 2013) demonstrated that SI could be a better criterion for early identification of ongoing hemorrhage, when compared to separated vital signs i.e., heart rate (HR) and systolic blood pressure (SBP) alone. Heidar et al. (Heidar et al. 2014) showed a significant association between baseline SI or SBP and the need for surgical intervention in patients with blunt abdominal trauma. However, the investigators found that the baseline HR was comparable in the surgical and conservative groups. Several scoring systems have been proposed for identification of post-traumatic hemorrhage and MT, however, most of them seemed to be complicated, resource-intensive, not validated, not immediately available, or time consuming (Callcut et al. 2016; DeMuro et al. 2013). Vandromme et al. (Vandromme et al. 2011) grouped their study cohort into 6 categories based on SI. The authors observed a linear relationship between SI and blood requirements with a 1.6 fold increased odds of massive transfusion for SI > 0.9–1.1 which substantially increased to 5.57 fold in patients with greater SI (> 1.1–1.3). This finding showed that SI is sensitive to changes in the circulating blood volume, and is useful in accurately predicting the need for early intervention to stop ongoing bleeding (Birkhahn et al. 2005).

To date, several approaches have been proposed to detect the extent of hypovolemic shock during early hemostatic resuscitation in trauma patients with variable applicability (Mutschler et al. 2013; Brockamp et al. 2012). In a validation study of 6 scoring systems for the need of MT after trauma, the greater overall precision was identified for the Trauma-Associated Severe Hemorrhage (TASH) score and Prince of Wales Hospital/Rainer (PWH) score (Brockamp et al. 2012). In addition, the Traumatic Bleeding Severity Score (TBSS) has been introduced to accurately predict the need for MT, but it had a sophisticated calculation that limits its use in the pre-hospital settings and/or upon hospital admission (Ogura et al. 2014). The ABC scoring system does not involve laboratory tests in its calculation and provides valuable information for the blood requirements in critical conditions. However, it requires FAST result which restricts its ready availability on ED admission except in well-designed ED of trauma centers where ultrasound machines are immediately available at bed side. Also, the use of other scoring systems that need laboratory tests and cumbersome calculations is inapplicable in emergency situations (Vandromme et al. 2011; El-Menyar et al. 2019). Recently, Motameni et al. concluded that in comparison to the clinical evaluation, the ABC criteria may overestimate the need for MT and it may even increase the product wastage, however, it could lead to earlier MTP activation (Motameni et al. 2018). In our study, ABC scores showed a significant correlation with SI (*r* = 0.62), a finding that supports the importance of SI as a simple alternative predictor in patient with SOI.

## Limitations

Our study has potential limitations due to its retrospective nature, and thus there is a possibility of missing information and inherent selection bias. Data were collected from a single center which would affect, in addition to the previous factors, the generalizability of our results. We focused on the SOI as it is a main source of bleeding in traumatic abdominal injury. The administration of blood products and massive transfusion is based on clinical judgment rather than on the objective measurement of hemorrhage. We lack information regarding the use of home medications (i.e., beta blockers) which could also influence SBP and HR; however, we would not expect to find frequent comorbidities because our patients are young with a mean age 29 years. Females constituted only one tenth of the study cohort whereas the vast majority (89%) was males; therefore the influence of gender disparity in trauma care was not discussed. The vital signs and the SI used in this study were based on initial values; we did not have detailed information regarding the exact timing of measurement of vital signs apart from the fact that these were measured as initial vital signs (within the first 5 min post arrival). Findings based on a single measurement may differ from the average of multiple readings. Assessment of the SI at ED admission might be influenced by the pre-hospital care involving the administration of intravenous fluids, sedation, and/or the use of vasopressors. Pain and anxiety might also have an influence on SBP and HR and therefore on the SI. There was no specific prehospital protocol, different than the international consensus, to follow. We did not report on the EMS transportation time that may affect the initial vital signs on arrival, however, based on our previous work the median EMS time was 58 min (interquartile range 45–77 min) (Al-Thani et al. 2014). This study comprised of mainly blunt trauma (95%) cases; the cardiovascular responses in patients with blunt trauma may differ from those with penetrating injuries. Although SI is also a useful tool for triaging patients for improved outcomes and effective utilization of resources (El-Menyar et al. 2017; Khan et al. 2013; Heidar et al. 2014), we do believe that further prospective studies comparing utility of SI with the other scoring tools are needed in patients with potential SOI.

## Conclusions

Shock index is a simple, fast, and bedside physiological tool that can predict the need for MTP activation in blunt abdominal trauma, particularly in patient who sustained SOI who can benefit from early massive transfusion and intervention. Further prospective studies are needed to support our findings and to compensate for our study limitations.

## Data Availability

Not applicable

## Abbreviations

FAST: Focused abdominal sonography assessment
MTP: Massive blood transfusion protocol
SI: Shock index
SOI: Solid organ injury
